# Planar-defect-rich zinc oxide nanoparticles assembled on carbon nanotube films as ultraviolet emitters and photocatalysts

**DOI:** 10.1038/srep04728

**Published:** 2014-04-17

**Authors:** Yunqing Zhu, Xiaohua Zhang, Ru Li, Qingwen Li

**Affiliations:** 1 Key Laboratory of Nano-Devices and Applications, Suzhou Institute of Nano-Tech and Nano-Bionics, Chinese Academy of Sciences, Ruoshui Road 398, Suzhou 215123, China

## Abstract

Structural defects in zinc oxide (ZnO) nanoparticles are complex and hard to be controlled during the synthesis, however, diversifying the chemical and physical performances. Here we report a rapid and low-temperature deposition method to fabricate planar-defect-rich ZnO nanoparticles on freestanding and aligned carbon nanotube films, different from common treatments which remove structural defects as many as possible. The defect energy states are very close to the valence band of ZnO and serve as recombination centers for a nearly monochromatic ultraviolet luminescence within a wavelength range of 373–376 nm. The absence of point defects, especially of oxygen vacancies whose energy level is <1 eV below the conduction band, allows photoinduced electrons and holes to take parts in possible photocatalytic reactions rather than to recombine at the shallow energy levels of planar defects.

Zinc oxide (ZnO) is a wide band gap semiconductor with potential applications in optoelectronics, transparent electronics, and light emitting devices. Due to the inevitable defects or low degree of crystallization, its luminescence usually exhibits a ultraviolet (UV) emission together with a wide band in the visible region^1^,^2^. Efforts to enhance either the UV or visible emission of ZnO include the coating of other materials to adjust the band structure^3^,^4^,^5^,^6^ and the improvement of crystallinity to tailor the emission behavior^7^. Similar modulation on the electronic structure is also applied to enhance the photocatalytic performance of ZnO, among which metal ions doping is widely used^8^,^9^,^10^. However, there exist complicated factors like the electronic structure, dopant concentration, energy levels of dopants, and *d* electronic configuration of the ions that contribute to the photocatalytic activities^9^. This makes it rather challenging to fabricate high performance photocatalysts based on pure ZnO nanostructures.

Recently, attempts on ZnO/carbon nanotube (CNT) hybrid structures have also attracted considerable attention and shown that the presence of CNT can influence the growth and thus the structure of ZnO nanoparticles. For example, various morphologies including ZnO nanowires^11^ or branched nanorods^12^ on CNT surfaces, ZnO-beaded CNTs^13^, and coaxial heterostructures^14^ were synthesized by using atomic layer deposition^12^,^14^,^15^, RF magnetron sputtering and substrate heating^13^,^16^,^17^,^18^, or a solution based treatment^19^,^20^. These structures yielded an enhanced UV to visible emission ratio^12^,^15^,^17^,^18^,^19^ owing to the improved surface-plasmon mediated emission, new defect emission, or a good crystalline quality of ZnO. The presence of CNTs also enhanced the photocatalytic performance of ZnO by the efficient separation of photoinduced charge carriers^21^,^22^,^23^. Today, with the development of mechanically strong, flexible, and freestanding super-aligned CNT films^24^,^25^,^26^, especially through the so-called method of array spinning^27^,^28^,^29^,^30^, new ZnO/CNT hybrid structures can be developed by taking advantages from the super alignment. Such new hybrid is also expected to possess new luminescent and photocatalytic properties.

Here, we show that ZnO nanoparticles exhibit a large number of basal plane stacking faults rather than other structural defects when being deposited on super-aligned CNT films. The deposition can be rapidly performed at ambient air condition under a temperature of 170°C. The planar defects result in a nearly monochromatic UV emission at 373–376 nm owing to their shallow energy levels as recombination centers of photoinduced electrons and holes. The absence of point defects like oxygen vacancies allows a long lifetime of photoinduced charge carriers, corresponding to a high photocatalytic ability. The hybrid film also exhibits flexibility and superior stability. An annealing treatment in the presence of O_2_ can convert the planar defects into oxygen vacancies, and thus shift the luminescence into blue and decrease significantly the photocatalytic performance. The rapid and low-temperature deposition of planar-defect-rich ZnO can be well applied on other freestanding films which contain a large area of carbon *sp*^2^ network and high air permeability.

## Results and discussion

### Deposition and crystal structure of ZnO nanoparticles

**Figure 1:**
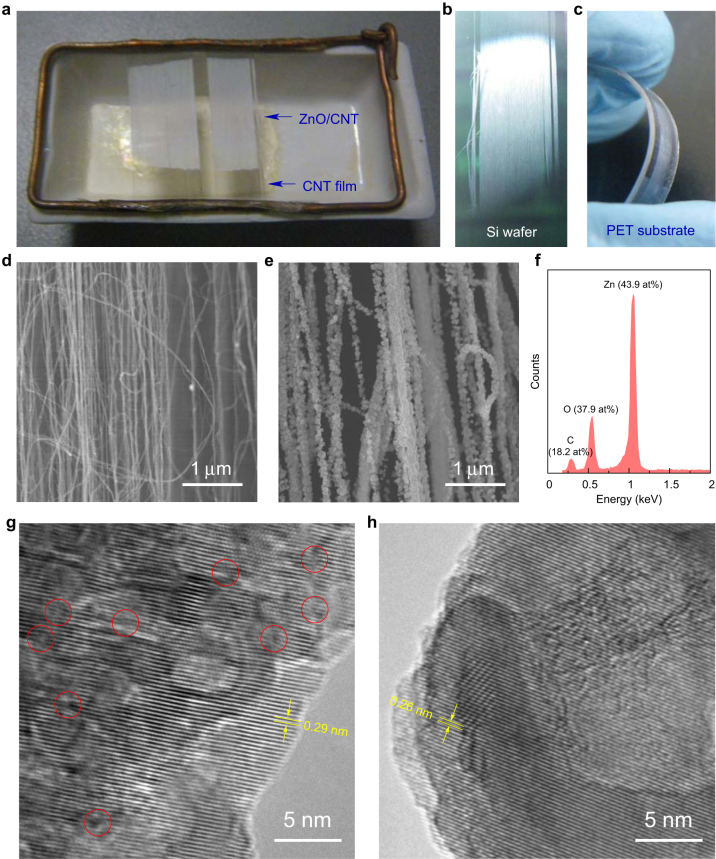
ZnO/CNT hybrid films. (a) ZnO nanoparticles deposited on freestanding CNT films. (b,c) ZnO/CNT films transferred onto different substrates with maintaining flexibility. (d,e) SEM images before and after the deposition. (f) EDX spectrum indicated that the ZnO nanoparticles were zinc-rich. The atom percentages are labelled in parentheses. (g,h) TEM images of ZnO nanoparticles before and after an annealing treatment. Several basal plane stacking faults in the as-produced structure are marked by red circles where the numbers of crystal planes entering and leaving the circles are not equal.

ZnO nanoparticles were deposited at 170°C on freestanding CNT films supported in a semi-opened ceramic boat by a thermal deposition using zinc acetylacetonate (Zn(C_5_H_7_O_2_)_2_) as source materials (Fig. 1a). The deposition temperature was determined by the thermogravimetric analysis of Zn(C_5_H_7_O_2_)_2_ (Supplementary Fig. S1). The as-produced ZnO/CNT hybrid film can be transferred onto a silicon wafer or a flexible polyethylene terephthalate (PET) substrate with maintaining the flexibility (Fig. 1b,c). Despite the uncoated regions owing to the limited source area at the bottom of the boat, the ZnO deposition was uniform in the coated region. Scanning electron microscopy (SEM) revealed that the ZnO-coated CNTs had a diameter of ~60 nm (Fig. 1e and Supplementary Fig. S2a,b). The CNTs were ~6 nm in diameter and had 2–3 walls^31^, see Supplementary Fig. S5a–c. Here, the ZnO thickness was controlled mainly by the mass of Zn(C_5_H_7_O_2_)_2_ (0.1 g as a default value) and slightly by the deposition time. Energy-dispersive X-ray spectroscopy (EDX) quantitatively showed a larger atom amount of zinc than oxygen, indicating that the defects were formed under the lack of oxygen (Fig. 1f).

Transmission electron microscopy (TEM) showed a certain level of crystallinity for the as-produced ZnO nanoparticles (Fig. 1g and Supplementary Fig. S2c,d). On one hand, the crystal planes were separated by 0.29 nm, a spacing close to but slightly larger than the idea value between the (002) planes. On the other hand, the crystal planes were not exactly planar as many curved segments were observed. This suggests that the ZnO nanoparticles were formed by stacking the imperfective (002) planes. The stacking faults were detected by TEM, where the numbers of crystal planes entering and leaving a small segment were not equal, corresponding to the missing or appearing of one plane (see the red circles in Fig. 1g and Supplementary Fig. S2c,d). An annealing treatment at 400°C was used to improve the crystallinity by healing the planar structural defects. This resulted in the decrease in the inter-plane distance (0.26 nm) and the flatness of the planes (Fig. 1h). Notice that the annealing did not heal all the defects; there were still a small number of stacking faults left (Supplementary Fig. S2e,f).

The crystal structure was further examined by X-ray diffraction (XRD) (Supplementary Fig. S3). The strongest XRD peak was observed at 2*θ* ≈ 34.4°, corresponding to the (002) plane, while the other two peaks for the (100) and (101) planes (2*θ* ≈ 31.7° and 36.2°) were weaker. This confirms that the preferential stacking direction was along the (002) plane. However, the (100), (002), and (101) peaks of the as-produced film appeared at 2*θ* about 0.1° smaller than their standards of 31.82°, 34.48°, and 36.30°, respectively, and was not sharp, corresponding to a high level of lattice disorder. This also agreed with the expanded distance of 0.29 nm between the (002) planes. Nevertheless, the XRD confirmed that the ZnO crystals were hexagonal wurtzite structure with lattice constants of *a* = 0.325 nm and *c* = 0.52 nm (JCPDS card No. 36-1451). After the annealing, as a large number of stacking faults were healed, the XRD peaks became higher and sharper, and reached the standards much more.

### Luminescent property of ZnO/CNT hybrid films

**Figure 2:**
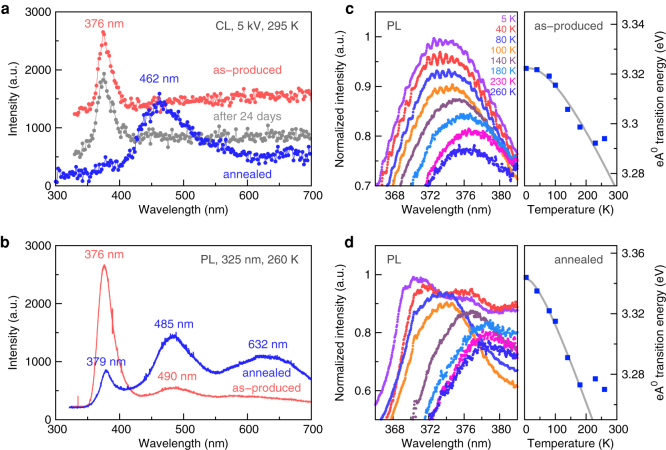
Luminescences of ZnO/CNT hybrid films. (a) Room temperature CL curves for one as-produced film, one kept at ambient conditions for 24 days, and another one after an annealing treatment. (b) PL curves at 260 K with a 325 nm incident laser for the hybrid structure before and after the annealing, respectively. (c,d) Temperature-dependent PL curves and the corresponding transition energies as a function of temperature, for the as-produced and annealed films, respectively. The intensities are normalized according to their highest values within the wavelength window of 360–390 nm, and are down-shifted for a better comparison. The gray curves are the fit results using the Varshni formula.

The presence of stacking faults had a strong influence on the luminescent property of ZnO. Cathodoluminescence (CL) measurement exhibited a single UV emission at 376 nm for the as-produced film at room temperature (Fig. 2a). This observation differed greatly from recent studies on ZnO nanostructures, where a multi-wavelength emission in the UV and visible regions were often observed^12^,^32^,^33^,^34^,^35^,^36^,^37^ and only few cases were concerning with the nearly pure UV emissions (wavelength ranging from 379 to ~400 nm)^15^,^18^,^19^. The nearly monochromatic UV emission was maintained without any change after the film was kept at ambient conditions with relative humidity <10% for 24 days. However, when the film was annealed, the CL spectrum became dominant blue where the emission peak was at 462 nm, indicating that the types of defect in ZnO had changed (Fig. 2a).

Slightly different from CL, photoluminescence (PL) measurement using a 325 nm laser exhibited a new emission peak at 490 nm for the as-produced film (Fig. 2b). This emission peak was attributed to another type of defect inside the ZnO nanoparticles, and was excited because photons travelled into ZnO deeper than a low voltage (5 kV) electron beam. After the annealing, this emission peak became much stronger and shifted to the wavelength of 485 nm, close to the emission at 462 nm in CL. Therefore we suspect that the annealing treatment converted the stacking faults into other types of defect, probably into those already existed inside the ZnO. There appeared also a new emission at 632 nm after the annealing, indicating that a third type of defect should exist and that this emission could arise from electron jumps between different defect energy states.

It was found that the luminescence had small dependence on the amount of ZnO deposited on a CNT film. By increasing the mass of zinc source from 0.1 g up to 0.2–0.35 g and using the same holding time of 30 min (see Methods), more ZnO nanoparticles were deposited around the CNTs. Fortunately, owing to the rapid deposition, there always existed planar structural defects in ZnO and the luminescence was still nearly monochromatic UV (Supplementary Fig. S4). Furthermore, as the ZnO particle size was much larger than the CNT diameter and the ZnO was deposited on isolated CNTs rather than bundled CNTs, the quenching effect by a metallic substrate was nearly negligible, corresponding to the high UV emission we have observed.

### Free-to-bound transition for UV emission

To identify the electronic nature of the UV emission and therefore to characterize the types of defect in ZnO, PL measurements were performed at temperatures ranging from 5 to 260 K (Fig. 2c,d). For the as-produced and annealed samples, the UV peaks both shifted to a smaller wavelength with decreasing the temperature. By fitting the temperature dependence of the transition energy, it is found that the UV emission was for a free-to-bound transition, i.e., the recombination of an electron from the conduction band with a hole bound to an acceptor state, an eA^0^ transition. The eA^0^ transition energy is given by 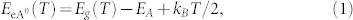
where *E_g_*(*T*) = *E_g_*(0) − *αT*^2^/(*T* + *β*) is the band gap for ZnO described by the Varshni formula^38^, and *E_A_* is the acceptor binding energy above the energy state of valence band. Here *E_g_*(0) is the band gap at *T* = 0, and *α* and *β* are two Varshni coefficients. The measurements were well fitted using equation (1) (see the right panels in Fig. 2c,d), giving *E_g_*(0) − *E_A_* = 3.322 eV, *α* = 3.56 × 10^−4^ eV K^−1^ and *β* = 220 K for the as-produced film, and 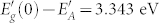, *α*′ = 6.44 × 10^−4^ eV K^−1^ and *β*′ = 85.3 K for the annealed one. Notice that the change in *E_A_* with temperature was ignored as 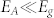, and also that the present measurements were not sufficient to estimate the value for either *E_g_*(0) or *E_A_*, while their difference was calculated. The increase in *α* means that the overall influence of thermal expansion and electron-phonon interaction on the fundamental band gap became much stronger after the annealing, as a result of the remarkable change in types of defect. As reflected in the UV emission, the change in *α* caused the different temperature dependence of the emission wavelength. For example, from 5 to 260 K, the UV emission redshifted from 373 to 376 nm for the as-produced film, but from 370 to 379 nm for the annealed one (Fig. 2c,d). The decrease in *β*, which is closely related to the Debye temperature, agreed with the observation that the low temperature fitting on 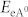 was valid at temperatures below 230 and 180 K for the as-produced and annealed films, respectively.

### Annealing-induced defect transition

**Figure 3:**
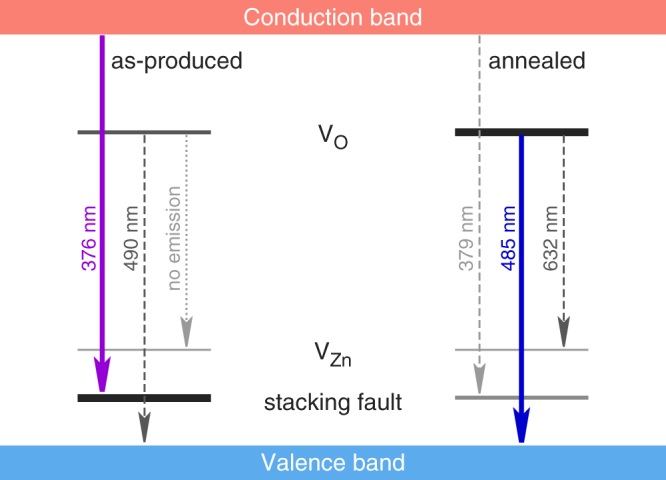
Schematic electronic level diagram of ZnO nanoparticles before and after the annealing treatment. The wavenumbers were taken from the PL measurements at 260 K. After the annealing, most stacking faults changed into V_O_, that converted the nearly monochromatic UV emission at 376 nm into a dominant blue emission at 485 nm.

The enhanced blue emission after the annealing arose from the newly formed defects. An energy analysis suggested the existence of another two possible defects of oxygen vacancy (V_O_) and zinc vacancy (V_Zn_), and one defect transition 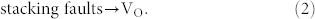
The transition took place as it was difficult to fully heal the stacking faults into perfect crystallinity by annealing, and thus a large number of V_O_ were formed after the treatment and served as centers of blue luminescence. It was confirmed by the enhanced blue emission in the annealed films. However, there should exist a small number of V_O_ deep inside the as-produced ZnO nanoparticles, as PL detected a small peak in the blue region while CL did not (Fig. 2a,b). The energy level of V_O_ was calculated to be 2.531 and 2.557 eV above the valence band, based on the wavelength of 490 and 485 nm at 260 K for the as-produced and annealed films. The existence of V_Zn_ was owing to the 632 nm emission for the annealed films, where electron jumps from V_O_ to V_Zn_ became possible owing to the increased number of energy states for V_O_. Therefore, all the observed PL emissions can be represented by a schematic electronic level diagram of the ZnO nanoparticles (Fig. 3).

As the defect types changed after the annealing, the light absorption might also be affected. From the UV-Vis spectra (Supplementary Fig. S5) one can find that the annealed ZnO/CNT hybrid films showed more absorption in the wavelength range of about 380–500 nm, owing to the increased V_O_. However, both the as-produced and annealed films showed intensive absorption in the UV band of about 230–365 nm, as such absorption was mainly caused by the ZnO bulk itself. The intensive UV absorption obviously has benefits to enhance the output luminescence signals.

### Photocatalytic degradation of acetaldehyde

**Figure 4:**
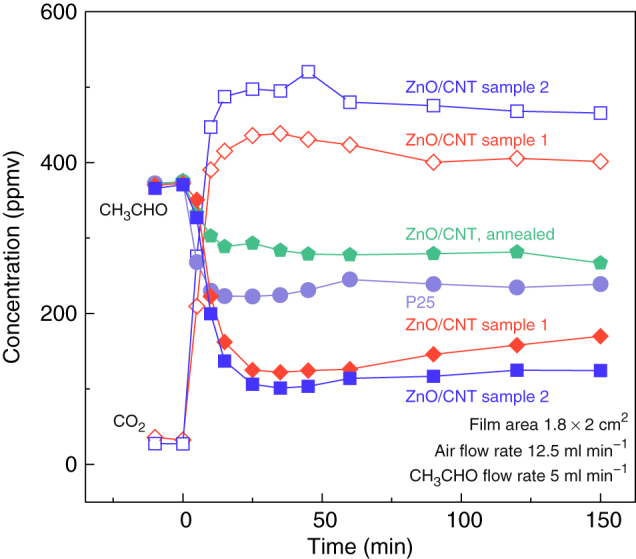
Photocatalytic degradation of acetaldehyde. Four samples were two ZnO/CNT hybrid films produced by using 0.2 and 0.35 g Zn(C_5_H_7_O_2_)_2_ as source materials, an annealed hybrid film (source mass 0.35 g), and commercial P25.

Besides the luminescent property, the photocatalytic degradation of acetaldehyde (CH_3_CHO) was investigated to show another possible application of the hybrid film. The degradation in gas phase was conducted in a tubular vessel reactor operating in a continuous flow mode^39^ and all the film samples were sized into 1.8 × 2 cm^2^ and transfered onto silicon substrates. By measuring the concentrations of CH_3_CHO and CO_2_, the conversion ratio and rate can be well estimated. For the hybrid film produced by using 0.2 g Zn(C_5_H_7_O_2_)_2_, the CH_3_CHO concentration decreased very quickly within 20 min from ~370 ppmv to 100–120 ppmv after turning on a 300 W Xe lamp (Fig. 4), corresponding to a conversion ratio up to 67–73%. By increasing the mass of Zn(C_5_H_7_O_2_)_2_ up to 0.35 g to increase the ZnO deposition, the photocatalytic reaction was further enhanced. In the meantime, the CO_2_ concentration increased by an amount nearly double the change in CH_3_CHO concentration. Similar to the stability of the luminescence, the photocatalytic ability did not decrease after being kept at ambient condition for weeks.

The photocatalytic ability of the hybrid films was compared with the commercial TiO_2_ nanoparticles (P25). Under the same test conditions, the CH_3_CHO concentration just decreased by ~150 ppmv, much less than the decreases by the hybrid films. More interestingly, although the sample area was nearly the same, the total mass of P25 we used was about 25 mg (the mass density of P25 is ~3.8 g cm^−3^), and was one order of magnitude larger than the masses of the hybrid films (both <2 mg). The small area density of the hybrid film (0.2–0.6 mg cm^−2^) was owing to the small number density of the superaligned CNT of 5–20 μm^−1^ (Fig. 1d). This means that the stacking-fault-rich ZnO nanostructures exhibited the much higher photocatalytic ability per unit particle mass than P25. Intuitively, this result seems strange because the shallow eA^0^ acceptor levels (Fig. 3) should reduce the catalytic activity when acting as the trapping or recombination centers for photoinduced electrons and holes. Fortunately, the eA^0^ acceptor levels were very close to the valence band and far from the conduction band. The large energy gap (*E_g_* − *E_A_* > 3.3 eV, more than 0.1 eV larger than the band gap of TiO_2_) allowed efficient reactions between the electrons with O_2_ and between the holes with organic compound, rather than the direct recombination via the hopping of electrons over such energy gap. Another result of the large energy gap was the significantly longer electron lifetime in ZnO than in TiO_2_^40^. In other words, the high photocatalytic ability thanks the lack of V_O_ in the as-produced samples, because the energy level of V_O_ was only <1 eV below the conduction band (Fig. 3) and preferred the recombination of electrons and holes. It was confirmed by the photocatalytic ability of the annealed hybrid films which only decreased the CH_3_CHO concentration by <100 ppmv (Fig. 4).

### Deposition mechanism of ZnO on *sp*^2^ surfaces

**Figure 5:**
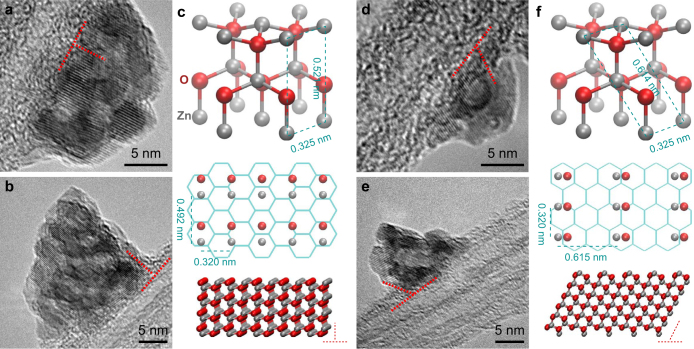
Mechanism of ZnO deposition on CNT surfaces. (a,b) TEM images of the perpendicular deposition. (c) Schematic illustration of the deposition of a (100) atom layer on the carbon hexagonal lattice. (d–f) TEM images and schematic illustration of the inclined deposition.

To analyze the deposition of ZnO nanoparticles, a procedure with zero holding time and 0.1 g zinc source was used (there was still a heating stage and cooling stage to allow a certain ZnO deposition, see Methods). The particle size was up to 20 nm (Fig. 5a,b,d,e and Supplementary Fig. S6). For most cases, the (002) crystal planes were perpendicular to the CNT surfaces (Fig. 5a,b and Supplementary Fig. S6c–f), while very few different cases were observed where the (002) planes had an angle of ~60° with the CNT surfaces. As the deposition method was seed layer free, the lattice matching between ZnO crystal and CNT *sp*^2^ network played the key role in determining the deposition procedure. The perpendicular deposition was suggested to be initialized by lattice matching between the (100) layer with the CNT surface (Fig. 5c). The ZnO lattice constant *a* = 0.325 nm is nearly 3/4 (76.3%) of the *sp*^2^ armchair period (3 × *a*_CC_ = 0.426 nm, *a*_CC_ the C-C bond length), and the constant *c* = 0.521 nm is only 5% larger than the double zigzag period (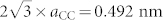). The perfect commensurability can maximize the van de Waals interactions between the (100) layer and the *sp*^2^ surface. For the inclined deposition, the formation of a (101) atom layer can be the most possible solution (Fig. 5f). The ideal lattice constants for the (101) plane are *a* = 0.325 nm and 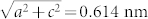, ~3/4 and ~5/2 of the armchair and zigzag periods, respectively. However, as the period along the zigzag direction becomes larger, the possibility of such inclined deposition is much smaller than the perpendicular one.

### Effect of deposition substrate

The rapid thermal deposition requires the freestanding substrate to be highly permeable to air. When a silicon substrate was used, ZnO nanoparticles was only sparsely found at the edges of the substrate. However, if the permeability is guaranteed, more ZnO nanoparticles can be deposited on various substrates. Two different examples are provided here. In one example, graphene oxide (GO) suspensions were coated on a small piece of screen window. After being dried at ambient condition, GO sheets with a lateral size close to 1 mm can be formed around the mesh points. The permeability of the screen window allowed the whole GO sheets covered by ZnO nanoparticles (Supplementary Fig. S7). The particle size was even one order of magnitude larger than the result on the aligned CNT films. In the second example, 4 layers of CNT films were stacked together as a substrate and ZnO nanoparticles were still easily deposited around the CNTs (Supplementary Fig. S7). This means that the deposition method can be widely applied on many different substrates.

Furthermore, CL characterization exhibited that the nearly UV emission was maintained for these two examples. Of great interest, the wavelength was 366 nm for the ZnO/GO hybrid structures, of 7–10 nm smaller than the ZnO/CNT hybrid films. It means that the level of defect on the *sp*^2^ surface also played an important role in determining the electronic level of the planar defects in ZnO. Notice that, when non-*sp*^2^ substrates were used, the luminescence differed greatly and monochromatic UV behavior no longer existed as many types of defects were formed in the as-produced structure.

In summary, the superaligned CNT films can be an exciting template to deposit planar-defect-rich ZnO nanostructures. The shallow eA^0^ acceptor levels of the planar defects, very close to the valence band, serve as recombination centers for a nearly monochromatic UV emission. The lack of V_O_ resulted in the high photocatalytic ability as the photoinduced carrier charges might have a long lifetime to allow them to react efficiently. The intelligent usage of planar structural defects and avoid of point defects open a new way in the design of multifunctional metal oxides.

## Methods

### Fabrication of hybrid films

The super-aligned CNT films were drawn out from vertically aligned CNT arrays synthesized by a chemical vapor deposition method^30^. Parameters concerning the growth, including the carbon source, catalyst, and growth temperature and time, were reported by our previous studies^31^,^41^. The film width ranged from 1 to 2 cm. The film was supported by a copper frame and was placed on a semi-opened ceramic boat. The zinc source Zn(C_5_H_7_O_2_)_2_ was placed inside the ceramic boat and below the CNT film. The default mass of Zn(C_5_H_7_O_2_)_2_ was 0.1 g, or others if specified. ZnO nanoparticles were coated on the CNT films in air by thermal deposition. The furnace temperature was increased from room temperature to 170°C at a heat rate of 8°C min^−1^, maintained at 170°C for 30 min (a holding time), and then automatically cooled down. To anneal the as-produced ZnO/CNT hybrid film, a gas flow of 300 sccm Ar was first introduced in to the furnace (3 inch in diameter) for 3 hour. Then the furnace temperature was increased to 400°C at a heating rate of 10°C min^−1^, maintained for 90 min, and cooled down to room temperature.

### Characterization

The structural characterization was performed with a Hitachi S-4800 field emission SEM (Hitachi Ltd., Tokyo, Japan) and an FEI Tecnai G2 F20 S-Twin TEM (FEI Corp., Hillsboro, USA). CL and PL were applied with a Gatan Mono CL3 system (Gatan Inc., Pleasanton, USA) and PI-PLE-2355/2558 + PIXIS-256E system (Princeton Instruments, Trenton, USA). A Bruker AXS D8 Advance XRD system (Bruker Corp., Karlsruhe, Germany) and an Oxford EDX detector (Oxford Instruments, Concord, USA) were also used to analyze the hybrid structure. The photocatalysis was performed with a CEL-GPPC continuous flow gas-phase reactor (Beijing CHN EDU AuLight Co. Ltd., Beijing, China) which contained a 300 W Xe lamp. The UV-vis absorption spectra were recorded in a Perkin-Elmer Lambda 950 spectrometer (PerkinElmer Inc., Waltham, USA). The gas concentrations were determined with an online gas chromatograph (SP7800, Keruida Technology Co. Ltd., Beijing, China). All of the photocatalyst samples were sized into 3.5–3.6 cm^2^. A mixture of air and CH_3_CHO was injected into a tubular vessel reactor. The flow rate of air and CH_3_CHO were 12.5 and 5 ml min^−1^, respectively. The gas pressure inside the vessel was 0.08 MPa.

## Author Contributions

Y.Z. carried out the experiments. R.L. grew the CNT materials and prepared the substrates. Y.Z. and X.Z. analysed the data. X.Z. and Q.L. wrote the manuscript. Y.Z., X.Z. and Q.L. contributed to the project planning.

## Supplementary Material

Supplementary Information Supplementary Information as one PDF
