# Supplementary material for: Planar-defect-rich zinc oxide nanoparticles assembled on carbon nanotube films as ultraviolet emitters and photocatalysts
Source: Sci Rep. 2014 Apr 17;4:4728. doi: 10.1038/srep04728 (PMC3989556; doi:10.1038/srep04728)
Supplement: Supplementary Information — as one PDF [file srep04728-s1.pdf]

Supplementary Information

Planar-defect-rich zinc oxide nanoparticles assembled on carbon nanotube films as ultraviolet emitters and photocatalysts

Yunqing Zhu, Xiaohua Zhang, Ru Li, and Qingwen Li

Key Laboratory of Nano-Devices and Applications,

Suzhou Institute of Nano-Tech and Nano-Bionics, Chinese Academy of Sciences,

Ruoshui Road 398, Suzhou 215123, China

Email: xhzhang2009@sinano.ac.cn; qwli2007@sinano.ac.cn

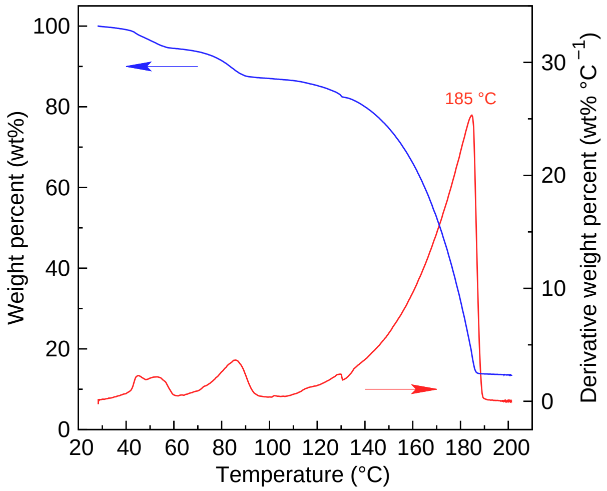

Supplementary Figure S1: Thermogravimetric analysis of $Zn(C_5H_7O_2)_2$. The weight loss occurred mainly during ∼150–190 °C.

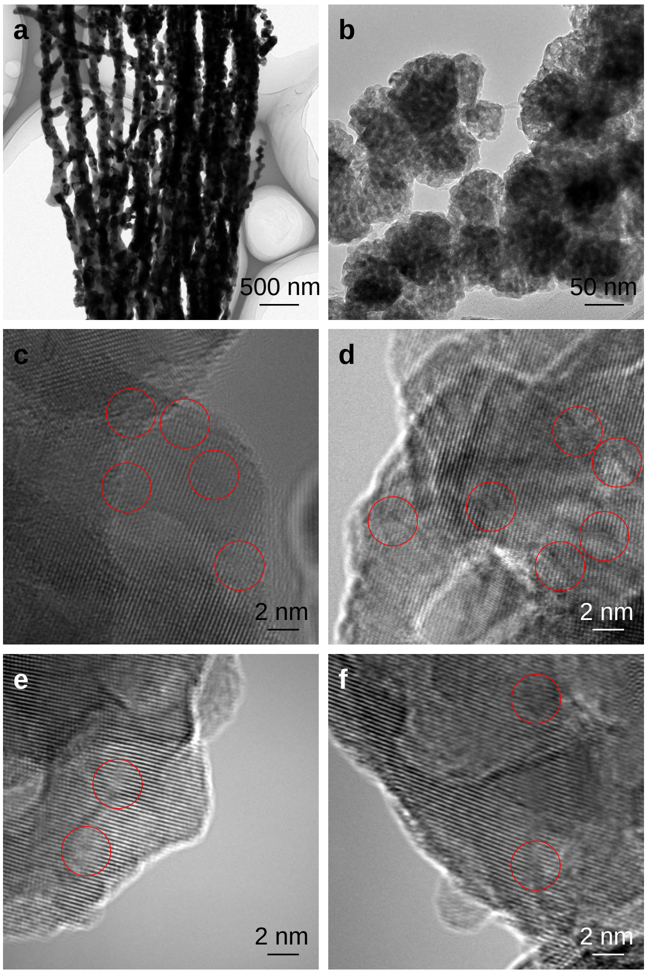

Supplementary Figure S2: TEM images of ZnO nanoparticles deposited on CNT films with the deposition temperature of 170 °C, zinc source mass of 0.1 g, and holding time of 30 min. a,b) The particle size of ZnO was about 60 nm. c,d) Planar structural defects widely existed in the as-produced samples, where the numbers of crystal planes entering and leaving a small segment were not equal. e,f) An annealing treatment healed remarkably the planar defects.

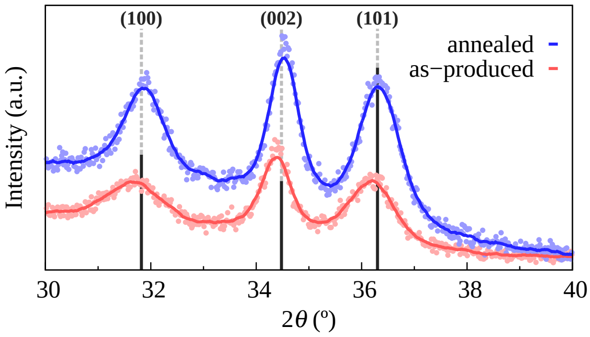

Supplementary Figure S3: XRD patterns of ZnO/CNT hybrid films before and after an annealing treatment. The (100), (002), and (101) patterns according to JCPDS card No. 36-1451 are provided for a comparison.

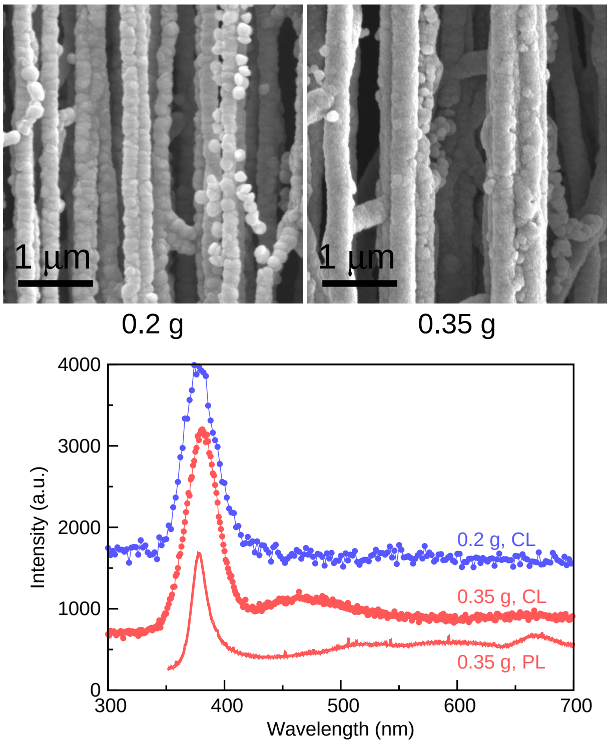

Supplementary Figure S4: Structure and luminescence characterization of ZnO/CNT hybrid films synthesized by using 0.2 g and 0.35 g $Zn(C_5H_7O_2)_2$, respectively.

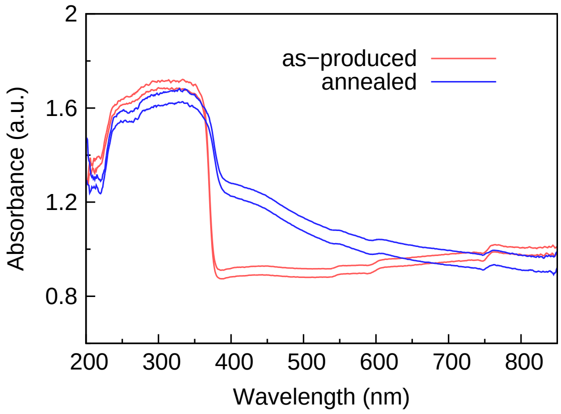

Supplementary Figure S5: UV-Vis spectra of ZnO/CNT hybrid films before and after an annealing treatment.

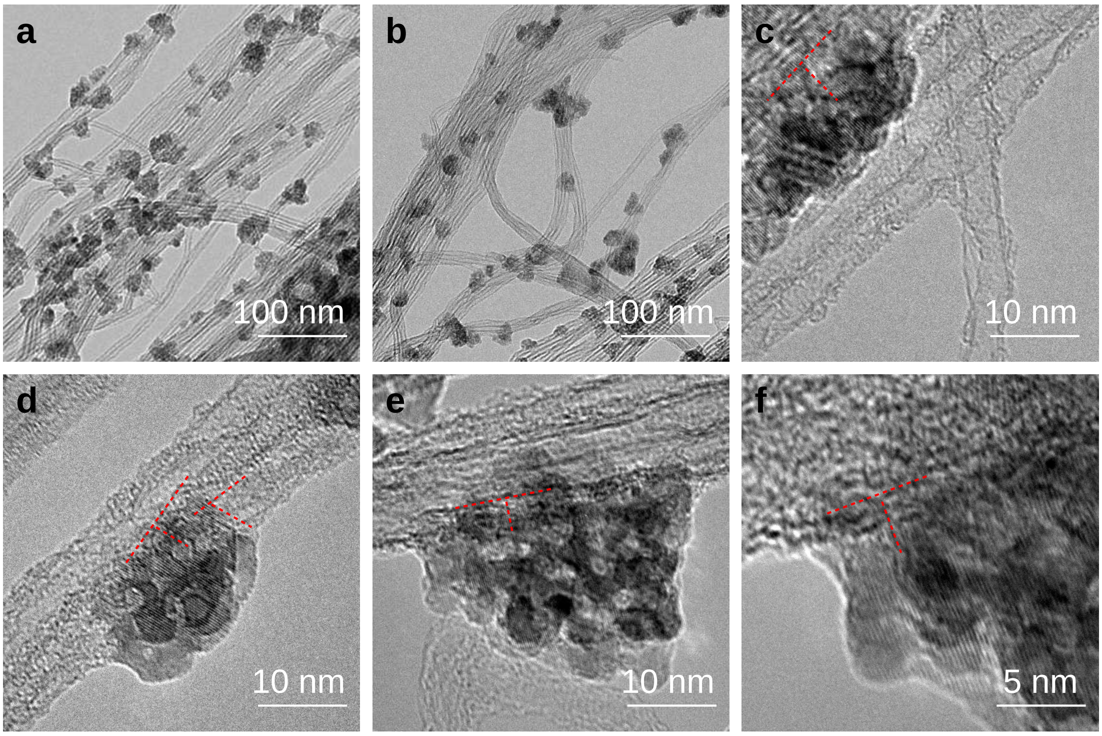

Supplementary Figure S6: More structure characterization of ZnO/CNT hybrid films synthesized by using 0.1 g $Zn(C_5H_7O_2)_2$ and zero holding time. a–c) ZnO nanoparticles did not cover all the CNT surfaces and thus it was possible to characterize the CNT structure. d) One case of inclined deposition of ZnO. c,e,f) Three cases of Perpendicular deposition.

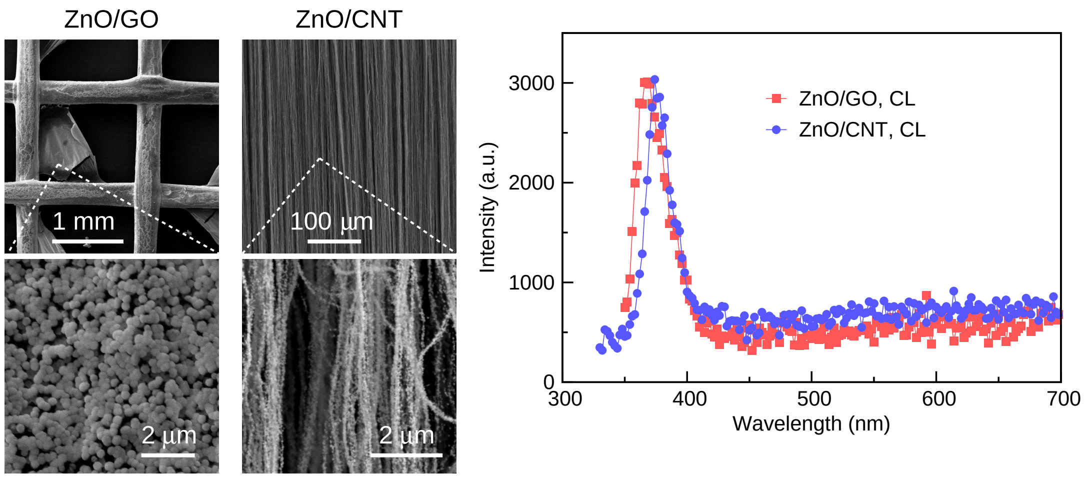

Supplementary Figure S7: Rapid and low-temperature assembly of ZnO nanoparticles on GO sheets and multi-layered CNT films, and the corresponding CL characterization.
